# Impact of healthcare worker shift scheduling on workforce preservation during the COVID-19 pandemic

**DOI:** 10.1017/ice.2020.337

**Published:** 2020-07-20

**Authors:** Dan M. Kluger, Yariv Aizenbud, Ariel Jaffe, Fabio Parisi, Lilach Aizenbud, Eyal Minsky-Fenick, Jonathan M. Kluger, Shelli Farhadian, Harriet M. Kluger, Yuval Kluger

**Affiliations:** 1 Department of Statistics, Stanford University, Stanford, California; 2 Program of Applied Mathematics, Yale University, New Haven, Connecticut; 3 Yale Cancer Center, New Haven, Connecticut; 4 School of Medicine, Yale University, New Haven, Connecticut; 5 Section of Infectious Diseases, Department of Medicine, Yale University, New Haven, Connecticut; 6 Section of Medical Oncology, Department of Medicine, Yale University, New Haven, Connecticut; 7 Department of Pathology, Yale University, New Haven, Connecticut

## Abstract

Reducing severe acute respiratory coronavirus virus 2 (SARS-CoV-2) infections among healthcare workers is critical. We ran Monte Carlo simulations modeling the spread of SARS-CoV-2 in non–COVID-19 wards, and we found that longer nursing shifts and scheduling designs in which teams of nurses and doctors co-rotate no more frequently than every 3 days can lead to fewer infections.

As the coronavirus disease 2019 (COVID-19) pandemic continues, healthcare workers (HCWs) report for duty, caring for both COVID-19 patients and patients with non–COVID-19 conditions. Reports from China and Italy suggest that HCWs are highly vulnerable to COVID-19 infection: in Italy, 20% of HCWs became infected with severe acute respiratory coronavirus virus 2 (SARS-CoV-2) at the peak of disease spread.^1^ Preventing COVID-19 infections among HCWs is critical for their safety and for stability of the healthcare delivery system. This includes stable functioning of non-COVID-19 wards, where HCWs may be exposed to SARS-CoV-2–infected patients who may not have undergone testing due to low clinical index of suspicion.

One approach to reducing infection rates is to optimize staff scheduling to minimize interactions between different HCWs and limit the patient pool to which HCWs are exposed. Despite reports of nosocomial infections, infection of HCWs by patients, and transmission of SARS-CoV-2 from one HCW to another, little is known about the effects of HCW team structure on hospital transmission of SARS-CoV-2.^2,3^ Experience from other pandemics is not necessarily applicable because infection and fatality rates differ. Therefore, we ran Monte Carlo simulations to explore various staffing possibilities with the goal of identifying staffing structures to minimize infections among HCWs on non–COVID-19 wards. For COVID-19 wards, in which the rate of patient-to-HCW transmission depends on personal protective equipment (PPE) and types of procedures and patient encounters, alternative input parameter choices for such simulations are needed; here, we solely address staffing in non–COVID-19 wards.

For the 5 scheduling designs represented in Figure 1, we simulated the spread of SARS-CoV-2 in hospital wards with various choices of model input parameters. The universal model parameters for COVID-19 included incubation period distribution (time from exposure to first symptom) and latent period distribution (time from exposure to becoming infectious.) Situation-dependent COVID-19 model parameters included preadmission infection probability of an admitted patient, team member infection probability at start of simulation, physician-to-patient, nurse-to-patient, patient-to-physician, patient-to-nurse, and HCW-to-HCW transmission probabilities, team-member days of absence after symptom onset, daily SARS-CoV-2 exposure probability of team members (eg, via elevator use, exposure to other staff), length of patient stay after showing COVID-19 symptoms, and length of simulation time. Model parameters that varied by hospital setting and service type included average team patient census, average patient hospitalization length, and the number of physicians and nurses on a team and on duty at all times. Parameters relevant to patient infectivity and patient acuity are discussed in Appendix B (online) and the model is described in further detail in Appendix C (online).

**Fig. 1.**
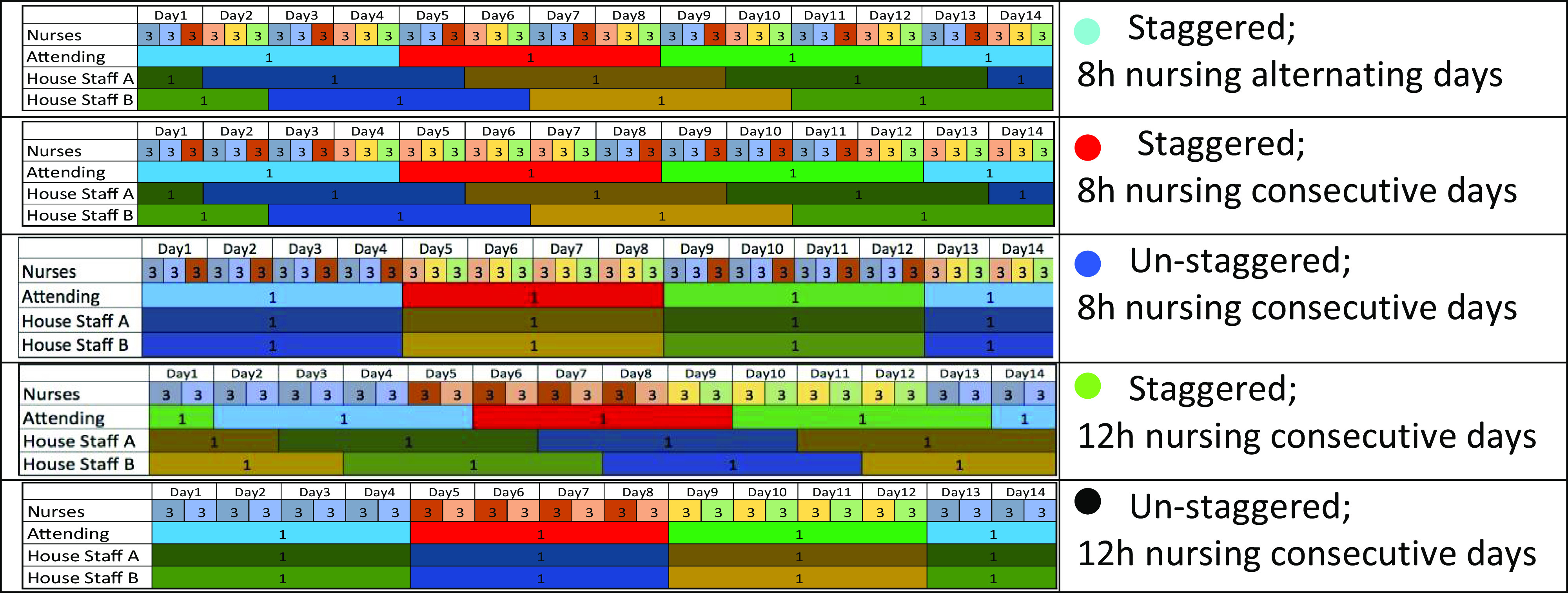
Scheduling designs. Schematic diagrams of 5 different scheduling designs for a team of 18 nurses, 3 attending physicians and 6 house staff. These diagrams correspond to the scenario in which physicians rotate every 4 days, and when applicable, cohorts of nurses rotate every 4 days as well. Each physician is represented by a unique color. In each shift there are 3 nurses (triplet). The identity of the nurses in each triplet is fixed as long as all nurses in the triplet are healthy. Each triplet is represented by a unique color. The right column describes the different scheduling designs. The colors of the bullet points are matched with the colors representing the scheduling designs of Figure 2. In Appendix A (online), details are provided for how the schedule for each design is adjusted when a HCW becomes ill and needs to be replaced.

To illustrate how scheduling decisions affect infection rates, we simulated 2 hospital teams, each including 6 house staff or advanced practice providers (APPs) and 3 attending physicians, 2 house staff and APPs, and 1 attending physician on rotation at a time (Fig. 1). The first team had 30 nurses (5 per shift), and the second team had 18 nurses (3 per shift). The average number of patients was set to 15 per day (5 per nurse or 3 per nurse, in settings with different patient acuity). Under normal circumstances, personnel rotations are staggered to ensure continuity of care and broad exposure for trainees to attending physicians and patients to enhance their educational experience. Rotation duration is also geared toward minimizing HCW fatigue. In a pandemic, these factors are considerably less important than HCW preservation. We compared scheduling options to minimize team failure, defined as the event that at some point there are insufficient attending physicians or house-staff/APPs to staff a fully functioning floor or insufficient healthy nurses to limit weekly hours to 48. Under all scenarios modeled, each nurse works an average of ≤36 hours per week. Figure 1 illustrates 5 staff scheduling designs for a team of 18 nurses, 3 attending physicians and 6 house staff with physician rotation durations of 4 days. Figure 2 depicts the outcomes of the 5 staff scheduling scenarios for mean patient hospital stays of 2 and 5 days, typical for maternity and medicine floors, respectively, indicating team failure probability as a function of physician rotation length. We simulated situations in which cohorts of nurses corotate with physician rotations compared to nursing schedules that were independent of physician schedules.

**Fig. 2.**
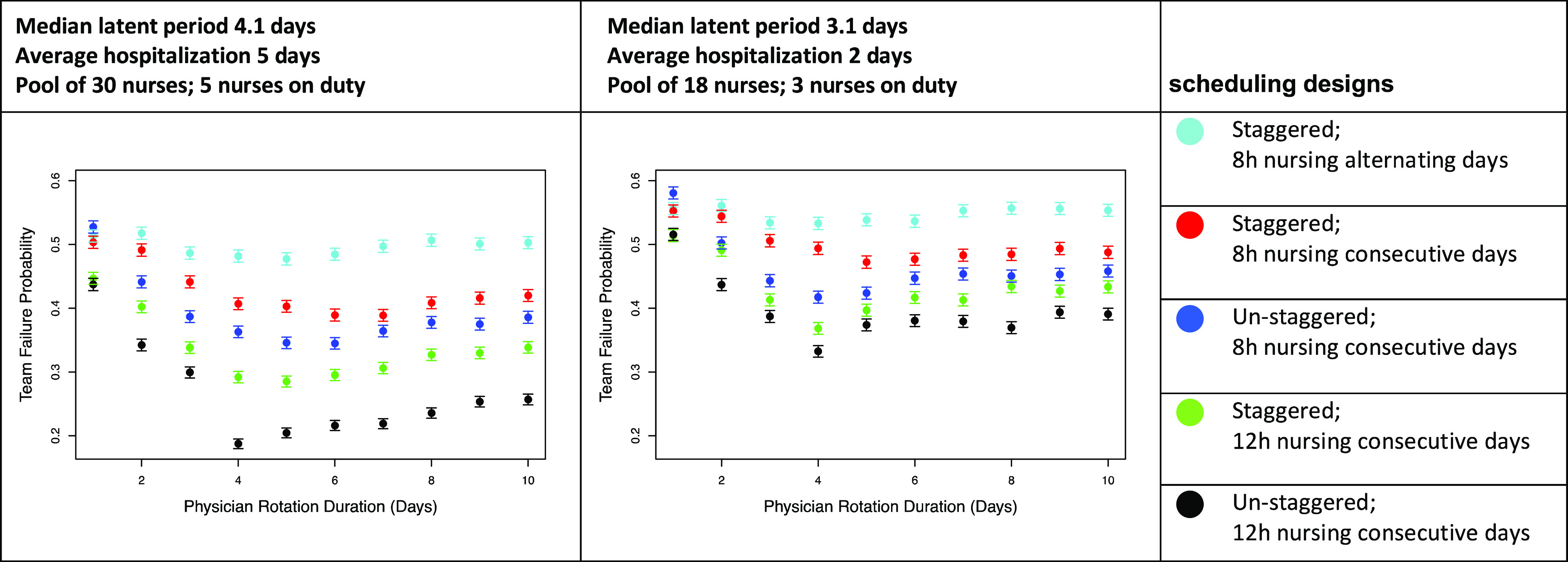
Probability of team failure versus physician rotation duration. Team failure probability is based on Monte Carlo simulations plotted by duration of physician rotation, modeled for a team caring for patients with 5-day average hospitalizations with fewer patients per nurse, such as internal medicine wards (left) or for patients with 2-day average hospitalizations and more patients per nurse, such as maternity wards (right). The plots compare the probability of team failure for 5 different scheduling designs. The designs simulated vary by whether they are staggered versus un-staggered, whether they have 8-hour nurse shifts or 12-hour nurse shifts, and whether nurses work consecutive days or work alternating days. In our simulations with nurses working consecutive days, when the physician rotations are sufficiently short, the nurses work the same number of consecutive days as the physician do. However, if the physician rotations are too long, the nurses are scheduled to work as many consecutive days as possible without exceeding 48 hours of work in the span of 1 week, and without exceeding 36 hours per week on average. Notably, due to unknown variables in the model, these plots do not suggest that the actual probability of team failure lies in the 20%–60% range, but rather, the plots are intended to demonstrate the relative improvement of various staff scheduling changes. From the plots above, and from similar plots that we generated with varying choices of the unknown parameters, we observe that scheduling designs with un-staggered rotations, 12-hour nursing shifts over consecutive days are favorable, and further, the probability of team failure is lower when all HCWs work at least 3–4 consecutive days.

Although the precise latent period of SARS-CoV-2 is unknown, the median incubation period is 5.1 days.^4^ COVID-19 patients are likely most infectious 24 hours before and 24 hours after first symptoms.^5^ Without frequent testing, shorter rotations increase the likelihood that infected HCWs will be off rotation for 24 hours before initiation of symptoms, while longer rotations expose fewer HCWs to the same infectious patient.

The rotation length that minimizes failure probability mainly depends on 2 factors: the median SARS-CoV-2 latent period, which is not precisely known, and the average hospitalization duration. Further understanding of the relationship between these factors is needed to make strong recommendations about optimal rotation length. However, in all simulations analyzed, physician and nurse rotation lengths of 1–3 days led to higher team failure rates; shorter rotations resulted in exposure of more HCWs to an infected patient. When the average patient stay is much longer than 5 days or when the median latent period is much shorter than 4 days, the benefit of un-staggering rotations decreased (data not shown). When patient stays were short, such as on maternity wards, the advantage of un-staggered rotations was consistent and universal across various parameters. Notably, because the actual probability of team failure is sensitive to other unknown parameters, plots such as those in Figure 2 should be used only to design optimal scheduling of shifts and not to forecast the actual probability of team failure. Our Rotation-Scheduler R code is available at https://github.com/KlugerLab/RotationScheduler.

In summary, pandemics necessitate widespread reassessment of workforce planning to ensure backup of sufficient uninfected HCWs. Using various input variables for our simulations for non–COVID-19 services, we make 3 primary observations: (1) Having all HCWs work at least 3 consecutive days reduces the chance of team failure, (2) longer nursing shifts (12 versus 8 hours) decreases the rate of HCW infection, and (3) avoiding staggering of rotations of attendings, house staff, and nurses reduces the number of infected HCWs. When applying this model to the real-world challenge of staffing hospital units, clinical setting variables such as trainee presence, patient acuity, stay length, and nurse–patient ratio will need to be considered. Similar modeling can be employed for teams treating known COVID-19 patients.

In conclusion, alternative staffing methods, in which groups of physicians and nurses share rotations that are at least 3 days long with 12-hour nursing shifts, should be considered for workforce preservation in the COVID-19 pandemic.

## Supplementary material

For supplementary material accompanying this paper visit http://dx.doi.org/10.1017/ice.2020.337. click here to view supplementary material
