# Supplementary material for: Impact of healthcare worker shift scheduling on workforce preservation during the COVID-19 pandemic
Source: Infect Control Hosp Epidemiol. 2020 Jul 20:1–3. doi: 10.1017/ice.2020.337 (PMC7403749; doi:10.1017/ice.2020.337)
Supplement: Supplementary file 1 [file S0899823X20003372sup001.docx]

**Appendix A: Schedule adaptations for sick healthcare workers (HCW)**

Figure 1 contains schematic diagrams that depict the original staggered and un-staggered schedules which are in place before any HCWs get sick. Here we describe in detail how the schedule adapts once a HCW gets sick. Let T_Nurse_ denote the length (in days) of each nursing rotation and let T_Physician_ denote the length of each physician rotation (in days.)

In the staggered setting, a healthy nurse is replaced after T_Nurse_ consecutive days of work, provided there is a healthy and available replacement. Similarly, a healthy physician is replaced after T_Physician_ consecutive days of work as long as there is a healthy and available replacement. It is worth noting that in the staggered setting, once a HCW becomes sick the offsets (in days) between the rotations of certain pairs of HCWs could change. For example, if attending A and house-staff member B have rotations that are originally offset by 2 days, it is possible that if one of them has to swap in for a sick physician they may end up with rotations that are offset by 1 day. It is even possible, though highly unlikely, for the schedule to become temporarily un-staggered, and even when this occurs the next HCW infection is likely to return the schedule to a partially staggered state.

In the un-staggered setting, every T_Nurse_ calendar days, all the nurses who are working and have a replacement are replaced. Similarly, every T_Physician_ calendar days, all the physicians who are working and have a replacement are replaced. Therefore, in the un-staggered setting, a replacement who joins a new group during the middle of that group’s rotation will not complete a full rotation and will be rotated out concurrently with members of the new group. The replacement, after being swapped out with the group after working only a partial rotation is not placed higher in the queue to be the next replacement, because the queue is based on the number of consecutive days of work/rest. However, a replacement nurse is indeed slightly more likely to be deemed “available” because they will be less likely to be poised to overwork beyond the cap of 48 hours in one week. It is worth noting that after a replacement occurs, the newly formed group of nurses is not likely to last forever—if the number of healthy available nurses is not divisible by the size of a nursing group, groups will not stick together in our simulation. When a nurse gets sick and eventually recovers, at some point the number of nurses in a group will not divide by the number of healthy nurses, and nursing groups will consequently be broken up.

**Appendix B: A discussion regarding accounting for patient infectivity and patient acuity**

Patient infectivity is important in modeling the chance of patient to HCW infection, and is driven by a number of complex factors such as time from initial infection, type of symptom, length of hospital stay, PPE, the type of interaction between patients and HCW, hospital air circulation, and type of treatment a patient receives (e.g. CPAP and other forms of respiratory support.) *.* Instead of modelling each of these factors that affect infectivity of patients separately, these factors are absorbed into the choice of two parameters in our model. The first relevant parameter is the probability that an infectious patient will transmit the virus to the nurse in a four-hour block of time in which that nurse is responsible for the patient, even if interacting with the patient intermittently (recall that in our simulations a nursing shift is either two four-hour blocks or three four-hour blocks). The second relevant parameter is the probability that an infectious patient will transmit the virus to the physician during a day in which that physician is responsible for the patient. When picking values for these two parameters, we assumed that PPE was unavailable. In light of these complex factors and the changing availability of PPE, the model was run for different choices of these two parameters, and all choices of parameters studied yielded results that were consistent with our final conclusions.

We assumed that infectivity did not vary with time (even though this is unlikely to be the case.) In particular, we assumed that once a patient becomes contagious they remain just as infectious until they are isolated and removed from the ward. While symptom onset is randomly generated for each infected individual from a lognormal distribution based on data from the literature, there are two relevant parameters in our model that control the duration in which an individual is infectious, using the onset of symptoms as a reference point. The first relevant parameter is the number of days between becoming infectious and the onset of symptoms (in figure 2, we explore scenarios in which this parameter is set to one or two days.) The other relevant parameter is the number of days after showing symptoms that a patient is infectious (we set this parameter to one day, not because this is the true value, but because we assumed that it would take about a day after showing symptoms for a patient to be diagnosed with COVID-19 and removed from the (non-COVID-19) ward or isolated appropriately.) It is worth noting that the most infective period based on virus shedding is believed to be 24 hours prior to and after presenting symptoms, and our choices of these two parameters reflect this information.

Patient acuity is reflected in a few of the parameters in our model. Two relevant parameters are the probability that an infectious patient will transmit the virus to the nurse and the probability that an infectious nurse will transmit the virus to a patient in a four-hour block of time in which that nurse is responsible for the patient, even if interacting intermittently with the patient. In settings with higher patient acuity, these parameters should be set to an appropriately higher value. Other model parameters, which reflect patient acuity and can easily be toggled in our model, are the average number of patients in the ward and the number of nurses that must be on duty at any given time. Currently, our model does not allow for the plausible scenario in which multiple nurses are treating the same patient simultaneously. As long as this is not the case, the algorithm can be used to run simulations for ICU wards.

**Appendix C: A detailed description of how the simulations were implemented**

We designed a simulator that can run many repeated trials for any given set of input parameters. In each simulation, the medical team and patient system evolves for 180 days or until the team fails. Team failure is defined as the event that there are not enough healthy and available medics to staff a fully functioning team for a day. We deem nurses unavailable if they have worked at least 48 hours in the previous seven days.

On day zero in our simulations, each HCW and each of the initial 15 patients has a 0.001 probability to be infected with SARS-CoV-2. Further, each HCW and patient who was infected by the virus on day zero, was randomly assigned an incubation period t_inc_ from a lognormal distribution with median 5.1, and was randomly assigned a number from a Uniform(0, t_inc_ ) distribution to randomly determine how many days into the infection, each infected person is on day zero of the simulation.

The simulation progresses each day in multiple stages:

***First stage:*** We simulate the daily transmission of the virus between HCWs and patients. In particular, each of the physicians interacts with all of the patients. When a patient and a physician interact if the patient is infectious (a person is infectious beginning one or two days prior to the onset of their first symptom) and the physician has not yet been infected, the probability of patient to physician transmission was set to 0.1. Similarly, when a patient and a physician interact, and the physician is infectious, the probability of transmission from physician to uninfected patient was set to 0.1.

Patient-nurse interactions are subsequently simulated in the same manner, with two key differences. First, the patients are partitioned into k groups, where k is the number of nurses on a shift simultaneously, and each nurse only treats patients in one of the k groups. Because nurses spend more time than physicians do with each individual patient, the probability of a transmission between a nurse-patient pair that sees each other was set to a higher value than that for a physician-patient pair. Second, the probability of nurse to patient transmission and patient to nurse transmission is appropriately adjusted based on duration of the nursing shifts (for example, the probability of no transmission from an infectious patient to a nurse that works 8 hour shifts will differ by an exponent of $\frac{3}{2}$from that for a nurse that works 12 hour shifts.)

Transmissions between HCWs are then simulated. To do this we compute N_eff_, the effective number of infectious HCWs. N_eff_ is calculated based on how many infectious HCWs are present and the number of hours each of those infectious HCWs works. Then we simulate whether or not each individual healthy HCW is infected by other HCWs with probability that depends upon both N_eff_ and the number of hours that this healthy individual HCW works.

In the simulations we looked at, we assume that patients do not transmit the virus directly to other patients as all patients are in separate rooms for hospitals we simulate. Including an option to simulate transmission in hospital wards where there is more than one patient per room is simple and does not require restructuring.

Finally, for each day of work, additional sources of infections can be introduced, such as infections in elevators, short interactions with other staff such as pharmacists, clerical staff and janitors, and infections outside of the hospital such as family exposure and supermarket runs. These affect each HCW with a small probability (less than 0.0001 per day in our simulations).

Note that for all of the simulated transmissions mentioned above, transmission is only assumed to occur to HCWs and patients who have not yet had the virus, and immunity is assumed once a HCW recovers from the virus. Finally, once all of the above transmissions are simulated, each newly infected patient and each newly infected HCW is assigned an independently drawn incubation period from a lognormal distribution with a median of 5.1 days.

***Second stage***: This stage of the simulation for the work day involves a random process for releasing and admitting new patients. After the daily transmissions are simulated, some patients will randomly leave and some new patients will be hospitalized. In particular, at the end of each day, each patient leaves with probability $\frac{1}{Average Patient Stay}$, and on average $\frac{Initial Number of Patients}{Average Patient Stay}$ new patients are hospitalized at the end of each day. This method of randomly accepting and discharging patients, gives us the desired average patient stay (an input parameter for our model.) The method also gives us a consistent average number of patients present equal to the initial number of patients (also an input parameter for the model.) Finally, we assume that patients who have exhibited COVID-19 symptoms for at least 24 hours are identified, isolated, and no longer seen by physicians and nurses on the team in person.

***Third stage:*** This stage of the simulation determines at each day which HCWs will work the following day. At the end of each day in the simulation (midnight), any HCW who began showing COVID-19 symptoms 21 days prior is either put in the category of “very ill” with probability 0.07 after which this HCW is unable to return to work, or is put in the category of “recovered” after which the HCW is able to return to work and is immune to the virus. In addition, at midnight, each HCW who started showing COVID-19 symptoms that day is replaced by the healthy HCW in the same category who has been waiting at home for the longest period of time. Further each nurse who has worked 48 or more hours in the past seven days is replaced by the nurse who has been off duty for the longest period of time among healthy nurses. If at least one of these replacements cannot be made, the team fails and the simulation ends.

In addition to replacing HCWs who start showing COVID-19 symptoms, physician and nursing rotations are also implemented in stage three. We have input parameters T_Physician_ and T_nurse_ to denote the number of consecutive days each physician and nurse is scheduled to work respectively. In the staggered setting, each HCW is replaced, whenever possible, after a physician works T_Physician_ days and after a nurse works T_nurse_ days. In the un-staggered setting, after every T_nurse_ days, all nurses who were on duty and can be replaced are replaced, and after every T_Physician_ days, all physicians who are on duty and can be replaced are replaced. Again, when implementing these rotations, HCWs are replaced by the healthy HCWs in the same category who have been waiting at home for the longest period of time.

After substituting symptomatic HCWs and fulfilling HCW rotations, the simulation progresses to the next day. The days in simulation progress until either the team fails or the 180 days completed without team failure.

So far, we described a single simulation. To estimate team failure probability for a given set of parameters and schedule design, we execute 10,500 simulations with the same parameter values. Team failure probability is estimated by computing the proportion of simulations that result in team failure.

Rotation-Scheduler was programmed in R. Rotation-Scheduler is available at <https://github.com/KlugerLab/RotationScheduler>. The code for all experiments is available on request and will be publicly available together with a detailed tutorial at https://github. com/KlugerLab/ Rotation-Scheduler-paper on publication.
